# Supplementary material for: Fire Acupuncture versus conventional acupuncture to treat spasticity after stroke: A systematic review and meta-analysis
Source: PLoS One. 2021 Apr 9;16(4):e0249313. doi: 10.1371/journal.pone.0249313 (PMC8034732; doi:10.1371/journal.pone.0249313)
Supplement: S1 Appendix — (DOCX) [file pone.0249313.s002.docx]

**S1 Appendix**

**Search strategy**

**Cochrane Central Register of Controlled Trials**

**#1  “Cerebrovascular Disorders” OR “Brain Ischemia” OR “Cerebral Hemorrhage” OR “Stroke” “Cerebrovascular” OR “Cerebrovascular Disorder” OR “cva”:ti,ab,kw**

**#2   MeSH descriptor: [Cerebrovascular Disorders]**

**#3   MeSH descriptor: [Brain Ischemia]**

**#4   MeSH descriptor: [Cerebral Hemorrhage] explode all trees**

**#5   MeSH descriptor: [Stroke] explode all trees**

**#6  1–5**

**#7  “ fire acupuncture” OR “‘warm acupuncture” OR “fire needle”:ti,ab,kw OR “‘Burn needle” OR “Red-hot needle”:ti,ab,kw OR “Fire-needle” OR “Fire needling”:ti,ab,kw OR “‘Fire acupuncture” OR “Fire-acupuncture”:ti,ab,kw**

**#8   MeSH descriptor: [Acupuncture] explode all trees**

**#9  MeSH descriptor: [Acupuncture Therapy]**

**#10  MeSH descriptor: [Electroacupuncture] explode all trees**

**#11  6 and 10**

**#12   “Muscle Spasticity” OR “Spasm” OR “Muscle Hypertonia” OR “Spasticity” OR “Muscle tightness” OR “Muscle stiffness” OR “Muscle pull”:ti,ab,kw**

**#13   MeSH descriptor: [Muscle Hypertonia] explode all trees**

**#14   12 or 13**

**#15   6 and 11 and 14 August 30, 2020**

**EMBASE**

**#1 ‘brain hemorrhage’/exp OR ‘brain infarction’/exp OR ‘brain ischemia’/exp OR ‘cerebrovascular accident’/exp**

**#2 ‘Cerebrovascular Disorders’:ab,ti OR ‘Brain Ischemia’:ab,ti OR ‘Cerebral Hemorrhage’:ab,ti OR ‘Stroke’:ab,ti ‘Cerebrovascular’:ab,ti OR ‘Cerebrovascular Disorder’:ab,ti**

**#3 1 or 2**

**#4 ‘ fire acupuncture’ OR ‘warm acupuncture’/exp**

**#5 fire needle:ab,ti OR ‘Burn needle’:ab,ti OR ‘ Red-hot needle’:ab,ti OR ‘Burn**

**needle’:ab,ti OR ‘ Fire-needle’:ab,ti OR ‘ Fire needling’:ab,ti OR ‘Fire**

**acupuncture’:ab,ti OR ‘ Fire-acupuncture’:ab,ti**

**#6 4 or 5**

**#7 'randomized controlled trial'/exp OR  ‘clinical trial’/exp**

**#8 ‘controlled clinical trial’:ab,ti OR randomized:ab,ti OR placebo:ab,ti OR randomly:ab,ti OR trial:ab,ti OR groups:ab,ti OR randomized controlled trial':ab,ti OR 'randomized':ab,ti OR 'placebo':ab,ti**

**#9   7 or 8**

**#10   ‘muscle hypertonia’/de OR ‘muscle rigidity’/exp OR ‘spasticity’/exp**

**#11 ‘Muscle Spasticity’:ab,ti OR ‘Spasm’:ab,ti OR ‘Muscle Hypertonia’:ab,ti OR**

**‘Spasticity’:ab,ti OR ‘Muscle tightness’:ab,ti OR ‘Muscle stiffness’:ab,ti**

**OR ‘Muscle pull’:ab,ti**

**#12   10 or 11**

**#13   3 and 6 and 9 and 12 August 30, 2020**

**MEDLINE/PubMed**

**#1   Fire needle[title/abstract]**

**#2   Burn needle[title/abstract]**

**#3  Red-hot needle[title/abstract]**

**#4  Fire-needle[title/abstract]**

**#5  Fire needling[title/abstract]**

**#6   Fire acupuncture[title/abstract]**

**#7   Fire-acupuncture[title/abstract]**

**#8 #1 or #2 or #3 or #4 or 5 or #6 or #7**

**#9 stroke [MeSH Terms] OR Cerebrovascular Disorders [MeSH Terms] OR Brain Ischemia [MeSH Terms] OR Cerebral Hemorrhage [Mesh] OR cerebral infarction [MeSH Terms]**

**#10 Cerebrovascular Disorders[Title/Abstract] OR Brain Ischemia [Title/Abstract] OR Cerebral Hemorrhage[Title/Abstract] OR Stroke[Title/Abstract] Cerebrovascular[Title/Abstract] OR Cerebrovascular Disorder[Title/Abstract]**

**#11 #9 or #10**

**#12 Paraparesis[MeSH Terms] OR Muscle Hypertonia [Mesh]**

**#13 spasticity[Title/Abstract] OR (Spasticity after Stroke[Title/Abstract])) OR (spastic after stroke[Title/Abstract]))) OR ((((Post-stroke spasticity[Title/Abstract]) OR (Spasticity after Stroke[Title/Abstract])) OR (spastic after stroke(Title/Abstract])) OR**

**Muscle Spasticity[Title/Abstract] OR Spasm[Title/Abstract]OR Muscle**

**Hypertonia[Title/Abstract] OR Spasticity[Title/Abstract] OR Muscle**

**tightness[Title/Abstract] OR Muscle stiffness[Title/Abstract] OR Muscle**

**pull[Title/Abstract]**

**#14 #12 or #143**

**#15 Randomized Controlled Trial [Publication Type] OR RCT [Publication Type] OR clinical trials[Publication Type]**

**#17 #8 and #11 and #14 and #15 August 30, 2020**

**China National Knowledge Infrastructure (CNKI) database .The following terms in Pingyin were used:**

**1. Huo Zhen (fire acupuncture)**

**2. Jia re Zhen (fire acupuncture)**

**3. Hong Re Zhen (fire acupuncture)**

**4. Shao Zhen (fire acupuncture)**

**5. 1 or 2 or 3 or 4**

**6. Zhong Feng (Stroke)**

**7. Nao Xue Guan Yi Wai (Cerebrovascular Disorders)**

**8. Jing luan (Spasticity)**

**9. Jing luan Zhuang Tai (Spasticity)**

**10. Ji Rou Jing Luan (Spasticity)**

**11. Pian Tan (hemiplegia)**

**12. Zhong Feng Hou Jing Luan (Post-stroke spasticity)**

**13 . Zhong Feng Hou Pian Tan (Post-stroke spasticity)**

**14 . 5 or 6 or 7 or 8 or 9 or 10 or 11 or 12 or 13**

**15. Lin Chuang Yun Yong (clinical application)**

**16. Lin Chuang Zhi Liao (clinical treatment)**

**17. Lin Chuang Yan Jiu (clinical research)**

**18. Lin Chuang Guan Cha (clinical observation)**

**19. Lin Chuang Dui Zhao (clinical comparison)**

**20. 14 or 15 or 16 or 17 or 18 or 19**

**21 5 and 14 and 20 August 30, 2020**
