# Supplementary material for: Fire Acupuncture versus conventional acupuncture to treat spasticity after stroke: A systematic review and meta-analysis
Source: PLoS One. 2021 Apr 9;16(4):e0249313. doi: 10.1371/journal.pone.0249313 (PMC8034732; doi:10.1371/journal.pone.0249313)
Supplement: Attachment — Submitted filename: Response to Reviewers.docx [file pone.0249313.s003.docx]

1.Please ensure that your manuscript meets PLOS ONE's style requirements, including those for file naming.

----Modified according to the magazine format

2. Please include the date(s) on which you accessed the databases or records to obtain the data used in your study

----Already provided in supplementary materials

3. In your PRISMA checklist, please provide the page numbers where the indicated information can be found

----Already provided in PRISMA checklist

4. Please include a separate caption for each figure in your manuscript.

----Already provided in all figure and table

5. Please include your tables as part of your main manuscript and remove the individual files. Please note that supplementary tables should be uploaded as separate "supporting information" files.

----Already include your tables as part of your main manuscript and remove the individual files.

**Reply to Major Revision**

**Reviewer #1**

**METHODS**

Page 6. First paragraph: after “PRISMA statement” the reference 12” must be enclosed in square brackets [12] I would like to know how the authors get the guiding question of this systematic review? Was an acronym used, for example PICO, or PICOS, or PICOT? If yes, please provide the structure and description and reference.

----I have added the references of (PRISMA) statement [16] and added the specific content of PICO (On page 5)

Page 6. Please to use MEDLINE/PubMed instead of PubMed as database.

----Has been revised, on page 3 and page 6

Page 6. Authors need to show more clearly the final key of the search strategy in each of the 8 databases accessed, mainly respecting the combinations of controlled descriptors (eg: MeSh Terms (MEDLINE / PubMed), emtree terms (EMBASE) ; etc ...) and intersections with keywords and synonyms using the Boolean AND & OR operators. I recommend that the authors present the search strategy in each database in the form of a table, containing the date on which the search was processed.

----A complete search strategy has been provided in the supplementary information section

Page 7. Data collection, extraction, and management.

Make it clear in the text when data were collected in the databases.
Another important point is to report how data was extracted (what information was extracted?)

-----The relevant content has been revised in the Data collection, extraction, and management section, on page 7

Page 7. Risk of bias assessment.
Please replace the Cochrane Bias Risk Assessment (RoB-1) to the recently updated and updated Cochrane Tool (RoB-2), since RoB-1 is coming into disuse.

------The relevant content has been modified in the Risk of bias assessment section, and table information has been added on pages 8 and 15-16

**RESULTS**

1.Table 1. Detail of studies include
Please, put the meaning of the second column (T / C) in the figure caption: (Treatment / Control group).

-----Modified in Table 1 and Table 2, on page 11 and page 13

2.Still in the second column "Age range" replaced to "Age range (M ± SD)" and put the meaning in the legend - mean and standard deviation.

-----Modified content in Table 1, on page 11

3.Check the studies Liu et al., 2018 and Chen et al., 2005, as there is no standard deviation. It is necessary to be consistent and maintain standardization.

-----Modified content in Table 1, on page 11

4.The study by Sheng et al 2017 the control group should be average age 56.21 instead og 5 6.21 (remove the space)

------Modified content in Table 1, on page 11

5.The study by Wang et al., 2018 the average age of the control group is misspelled. In the results on page 9 it is stated that ”The ages of the patients ranged from 34 to 80 years. Therefore, I believe that instead of C: 99.13 ± 33.86 it must be wrong. Please check.

--------Modified content in Table 1, on page 11

**DISCUSSION**

Page 17. Please, consider to include the reference doi:10.1177/2515690X19834169, together with the others [53,55].
“Interestingly, according to current research, acupuncture is a universally recognized non-drug treatment that can have a beneficial role in diseases that often accompany stroke, including depression, fatigue, and cognitive decline[53-55; Abrahão et al., 2019

-----Already added, in reference 60

**Reviewer #2**

1. Search terms need to provided in complete. "as an example is provided in the Supplementary material" is not acceptable. Search terms in Eight databases are different (PubMed, Web of Science, the Cochrane database, EMBASE, CBM, CNKI, WanFang, and VIP) Please attach syntax used in each database as supplementary.

------Has been added in the supplementary material section

2. Who are “two independent investigators”?

It has been explained in the section Data collection, extraction, and management, on page 7

3. It will be better to show kappa for the selection and data extraction. Please show the data of kappa of agreement during the systematic searches. How disagreements were solved during the systematic search among two independent reviewers?

------Has been added in the Risk of bias assessment section, on page 8

4.Figure1, suggest to use PRISMA 2009 Flow Diagram platform

------The format of the PRISMA 2009 Flow Diagram platform has been used, as shown in Figure 1.

5. Forrest plots and funnel plots need to be provided.

------A forest diagram has been added to all the meta results in the article, and the content of the funnel diagram has been added, as shown in Figure 12, pages 8 and 21

7. Random or Fixed effect was used, needs to be specified in the abstract.

-------Already added in the abstract section, on page 3

8.Authors should discuss the reason of heterogeneity.

-----Related content has been added in the discussion section, page 23

9. There is still a considerable heterogeneity as in your limitation. Meta-regression analysis is then strongly recommended.

------Meta-regression content has been added to the Data Synthesis section, and the results are calculated in Table 7, on pages 8 and 22

The PICOS of the meta-analysis should be clearly reported.

-------It has been explained in detail in the Methods section, on page 5
