# Supplementary material for: Fire Acupuncture versus conventional acupuncture to treat spasticity after stroke: A systematic review and meta-analysis
Source: PLoS One. 2021 Apr 9;16(4):e0249313. doi: 10.1371/journal.pone.0249313 (PMC8034732; doi:10.1371/journal.pone.0249313)
Supplement: Attachment — Submitted filename: Response to Reviewers.docx [file pone.0249313.s004.docx]

**Reply to Minor Revision:**

1.Abstract: Add in the “method” that the methodological evaluation or critical appraisal of the included articles was assessed using RoB-2. And in the results, to add that “according to the criteria of the RoB 2.0 tool, most of the studies are considered to have some concerns.

----Has been added, on page 3

2.METHODS
Data collection, extraction, and management: write the phrase in the past instead of using the verb in the future.
Data syntheses: Same. Please use the past tense “Random effect model were used….”

----The content status of the corresponding part has been modified, on page 6-7

3.RESULTS
  Table 5. Please to replace the symbols “+/-” by the official of RoB-2
  Low (+)
  High (-)
  Some concerns (?)
  Also the column “Overal risk” shoud be presenting as the last column in this Table.

----Table 5 has been modified as required
